# ﻿Taxonomy of *Buelliaepigaea*-group (Caliciales, Caliciaceae), revealing a new species and two new records from China

**DOI:** 10.3897/mycokeys.92.83939

**Published:** 2022-08-05

**Authors:** Min Ai, Li Juan Li, Fiona Ruth Worthy, An Cheng Yin, Qiu Yi Zhong, Shi Qiong Wang, Li Song Wang, Xin Yu Wang

**Affiliations:** 1 Key Laboratory for Plant Diversity and Biogeography of East Asia, Kunming Institute of Botany, CAS, Kunming, Yunnan 650201, China Kunming Institute of Botany Kunming China; 2 Yunnan Key Laboratory for Fungal Diversity and Green Development, Kunming Institute of Botany, CAS, Kunming, Yunnan 650201, China Senckenberg Research Institute Frankfurt am Main Germany; 3 Department of Botany and Molecular Evolution, Senckenberg Research Institute, 60325 Frankfurt am Main, Germany Kunming Institute of Botany Kunming China; 4 Kunming Botanical Garden, Kunming Institute of Botany, CAS, Kunming, Yunnan 650201, China Senckenberg Research Institute Frankfurt am Main Germany

**Keywords:** Lichenized fungi, nuITS-nuLSU-mtSSU-β-tubulin, phylogenetic analysis, terricolous, Tibetan Plateau

## Abstract

During the Second Tibetan Plateau Scientific Expedition and Research Program, we discovered that white terricolous lichenized fungal species of *Buellia* De Not. were widely distributed across the Tibetan Plateau. After examining their morphology, chemistry and phylogeny, we describe *Buelliaalpina* Xin Y. Wang & Li S. Wang, **sp. nov.** as new to science. It is present in alpine meadows, and is characterized by its effigurate thallus, distinct linear marginal lobes, cover of thick white pruina and four-spored asci. This is also the first report of *Buelliaelegans* Poelt and *Buelliaepigaea* (Pers.) Tuck from China. The *Buelliaepigaea*-group has previously been characterized by white and often effigurate thalli that occur mainly on soil. However, our results show that species in this group actually belong to two distinct clades. This conclusion is based on analyses of the nuITS region and the combined regions dataset (nuITS-nuLSU-mtSSU-β-tubulin). We discuss differences in morphology, anatomy, chemistry and ecology among the putative *Buelliaepigaea*-group. Detailed descriptions and figures for the three species from China and a key for species of *Buelliaepigaea*-group are provided.

## ﻿Introduction

The lichen genus *Buellia* De Not. (Caliciales, Caliciaceae) comprises approximately 400 species worldwide (Bungartz et al. 2007). *Buellia* s.l. is characterized by a crustose thallus, black lecideine apothecia, *Bacidia*-type asci, brown ascospores with one or more septate and reddish-brown or rarely hyaline hypothecia. Several other genera, which were previously included in *Buellia* s.l. (such as *Amandinea* M. Choisy, *Diplotomma* Flot. and *Tetramelas* Norman), have since been segregated based on their morphology, anatomy, chemistry and ecological environment (Scheidegger 1993; Marbach 2000; Nordin 2000). However, there remain other *Buellia* s.l. species with distinct morphological characters currently placed in groups instead of defined genera. Two examples are the species related to *Buelliaaethalea* (Ach.) Th. Fr. or *B.epigaea* (Pers.) Tuck, which are currently treated as *aethalea*-group or *epigaea*-group, respectively (Poelt and Sulzer 1974; Scheidegger 1993). Their current classification is based solely on external morphology, without the support of molecular data, therefore strict phylogenetic relationships between *Buellia* s.l. species remain unclear.

More than 64 species of the genus *Buellia* s.l. were previously reported from China, mostly located in the Tibetan Plateau region (Wei 2020; Wang et al. 2020). During the Second Tibetan Plateau Scientific Expedition and Research Program (STEP), a large number of additional lichen specimens were collected, including *Buelliaepigaea*-group species.

The *Buelliaepigaea*-group contains seven species; these are characterized by white and often effigurate thalli that occur mainly on soil. *Buelliaepigaea*, which was reported from Europe, is the core species of this group. *Buelliaasterella* Poelt & Sulzer and *B.elegans* were reported from Europe, and *B.* z*oharyi* Galun was reported from Asia and Europe by Poelt and Sulzer (1974). Trinkaus and Mayrhofer (2000) published a revision of the four species above. A further three new species were described from Australia: *Buelliadijiana* Trinkaus, *B.georgei* Trinkaus, Mayrhofer & Elix, and *B.lobata* Trinkaus & Elix (Trinkaus et al. 2001). *B.epigaea*, *B.dijiana* and *B.georgei* formed a monophyletic clade, but data is still lacking for the remaining species (Grube and Arup 2001).

The aim of this study is to determine which *Buelliaepigaea*-group species are distributed in China and whether they form a monophyletic clade. For this purpose, we carried out a phylogenetic study of the *Buelliaepigaea*-group based on four loci.

## ﻿Materials and methods

### ﻿Morphological and chemical analyses

During STEP, 92 specimens of the *Buelliaepigaea*-group were collected from the Qinghai-Tibetan Plateau and deposited in the Lichen Herbarium, Kunming Institute of Botany, China (KUN). Morphological characteristics of thalli and apothecia were examined under a dissecting microscope (Nikon SMZ 745T). Anatomical characteristics of apothecia were examined under an optical microscope (Nikon Eclipse Ci-S). Photographs were taken using a digital camera (Nikon DS-Fi2). Descriptions of the range of anatomical characteristics for each species were determined by the smallest and largest single values measured for all specimens. Thin-layer chromatography (TLC) was performed in order to identify secondary metabolites using solvent systems C (toluene: acetic acid = 85:15), according to Orange et al. (2001).

### ﻿DNA extraction, amplification and sequencing

DNA was extracted from fresh apothecia or thallus pieces with a DNA secure Plant Kit (TIANGEN) according to the manufacturer’s instructions. Amplified gene markers and their corresponding primers are shown in Table 1. PCR amplifications were achieved using 1.1 × T3 Super PCR Mix (TSINGKE) in a 25 µL total volume, containing 1 µL of genomic DNA, 1 µL of a 10 mM solution for each primer and 22 µL of 1.1 × T3 Super PCR Mix. The PCR program was: initial denaturation at 98 °C for 3 min, followed by 35 cycles of 98 °C for 10 s, 54–56 °C for 10 s, 72 °C for 15 s, followed by a final extension at 72 °C for 2 min. The PCR products were sequenced with the same amplification primers using Sanger technology by Tsingke Biotechnology Co., Ltd. (Kunming).

**Table 1.** Gene markers and primer pairs used in this study.

Gene markers	Primers	Sequences of Primers 5’-3’	References
nuITS	ITS1F	CTTGGTCATTTAGAGGAAGTAA	Gardes and Bruns 1993
	ITS4	TCCTCCGCTTATTGATATGC	White et al. 1990
nuLSU	LR0R	GTACCCGCTGAACTTAAGC	Rehner and Samuels 1994
	LR5	ATCCTGAGGGAAACTTC	Vilgalys and Hester 1990
mtSSU	SSU1	AGCAGTGAGGAATATTGGTC	Zoller et al. 1999
	SSU3R	ATGTGGCACGTCTATAGCCC	
β-tubulin	Bt3-LM	GAACGTCTACTTCAACGAG	Myllys et al. 2001
	Bt10-LM	TCGGAAGCAGCCATCATGTTCTT	

### ﻿Phylogenetic analyses

All newly obtained original sequences were edited manually using GENEIOUS v8.0.2. Their taxon name, voucher and GenBank accession number are shown in Table 2. All sequences, including those downloaded from GenBank, were aligned using MAFFT v7 with the option of E-INS-I (Katoh et al. 2005). Ambiguous regions were excluded using GBLOCKS (Talavera and Castresana 2007) with the default settings. Congruence between different gene regions was analyzed before combining. Bayesian inference (BI) and maximum likelihood (ML) were employed to determine the phylogenetic relationships. The best-fit partition substitution models were selected based on the lowest Bayesian information criterion (BIC) using PARTITION FINDER 2 (Guindon et al. 2010; Lanfear et al. 2012, 2017): nuITS dataset (TIM2e+G4) and the combined regions dataset (GTR+G for ITS1, ITS2 and mtSSU; GTR+I+G for 5.8S, nuLSU and β-tubulin), respectively.

**Table 2.** Specimens used in this study, with taxon name, voucher and GenBank accession number. Newly obtained sequences are in bold font. “*” indicates that the sample was not included in the combined regions’ dataset analysis. “NA” indicates that there is no sequence available.

Taxon	Voucher	Accession number			
		nuITS	mtSSU	nuLSU	β-tubulin
*Acoliuminquinans*	Wedin 6352 (UPS)	AY450583	AY143404	AY453639	KX529023
*Ac.karelicum*	Hermansson 16472 (UPS)	KX512897	NA	KX512879	NA
*Amandineapunctata* 1	18-60759 (KUN)	OL467351	NA	NA	NA
*Am.punctata* 2	AFTOL 1306	HQ650627.1	NA	DQ986756.1	NA
*Buelliaalpina*	**16-53720 (KUN)**	**OM914626**	NA	NA	NA
*B.alpina*	**16-53737 (KUN)**	**OM914627**	NA	**OP060154**	**OM925561**
*B.dijiana*	-	AF250788	NA	NA	NA
*B.disciformis* 1	EDNA09-01524	FR799139	NA	NA	NA
*B.disciformis* 2	EDNA09-02095	FR799136	NA	NA	NA
*B.disciformis* 3	EDNA09-02116	FR799138	NA	NA	NA
*B.elegans*	**18-60340 (KUN)**	**OM914622**	NA	**OM935566**	**OM925559**
*B.elegans*	**20-68266 (KUN)**	**OM914634**	NA	**OM935569**	**OM925562**
*B.elegans*	**XY19-272 (KUN)**	**OM914624**	NA	**OM935567**	**OM925560**
*B.elegans*	**18-59513 (KUN)**	**OM914623**	NA	NA	NA
*B.elegans	**18-62336 (KUN)**	**OM914630**	/	/	/
*B.elegans	**XY19-1907 (KUN)**	**OM914632**	/	/	/
*B.elegans	**XY19-1372 (KUN)**	**OM914631**	/	/	/
*B.elegans	**XY19-2308 (KUN)**	**OM914633**	/	/	/
*B.elegans	**12-34754 (KUN)**	**OM914625**	/	/	/
*B.elegans	**10-0089 (KUN)**	**OM914636**	/	/	/
*B.elegans	16-0084 (NXAC)	MN103116	/	/	/
*B.elegans	Beck 242 (GZU)	AY143411	/	/	/
*B.elegans	Leavitt 19085	MZ922074	/	/	/
*B.epigaea*	**XY19-1218 (KUN)**	**OM914628**	**OM913210**	**OM935568**	NA
*B.epigaea*	**XY19-2294 (KUN)**	**OM914629**	**OM913211**	NA	NA
*B.epigaea	-	AF250785	/	/	/
*B.epigaea	**18-59162 (KUN)**	**OM914635**	/	/	/
*B.georgei*	Trinkaus 356a (GZU)	AJ421416	NA	NA	NA
*B.zoharyi* 1	SA2	MG592314	MG592321	MG592328	MG592346
*B.zoharyi* 2	MT30	MG592315	MG592322	MG592329	MG592347
*B.zoharyi* 3	SA6	MG592316	MG592323	MG592330	MG592348
*B.zoharyi* 4	TE13	MG592317	MG592324	MG592331	MG592349
*Caliciumnobile* 1	Tibell 21968 (UPS)	KX512913	KX512988	KX529070	NA
*C.nobile* 2	Tibell 23396 (UPS)	KX512914	KX512987	KX529071	NA
*Diplotommaalboatrum* 1	**18-60034 (KUN)**	MN615696	OL467286	OL444781	**OM925557**
*Di.alboatrum* 2	**18-60448 (KUN)**	MZ224658	OL467287	OL444782	**OM925558**
*Di.venustum* 1	**18-58557 (KUN)**	OL467349	OL467284	OL444779	**OM925555**
*Di.venustum* 2	**18-58102 (KUN)**	OL467350	OL467285	OL444780	**OM925556**
* *Di.venustum* 3	XY19-252 (KUN)	OL467353	/	/	/
*Heterodermiaspeciosa*	Wetmore (S)	KX512927	KX512975	KX512868	KX529000
*He.vulgaris*	Frisch 11/Ug1226 (UPS)	KX512928	KX512989	KX512857	NA
*Phaeophysciaciliata*	Prieto (S)	KX512929	KX512958	KX512886	KX529012
*Ph.orbicularis*	Prieto 3012 (S)	KX512930	KX512967	KX512876	NA
*Physciaaipolia*	Wedin 6145 (UPS)	KX512931	AY143406	AY300857	KX529021
*P.tenella*	Odelvik and Hellström 0827 (S)	KX512932	KX512974	KX512869	NA
*Pyxinecoccoes*	Prieto (S)	KX512936	KX512964	NA	KX529010
*Py.subcinerea*	-	HQ650705	NA	DQ883802	NA
*Py.sorediata*	Wetmore 91254 (S)	KX512937	KX512973	KX512870	KX529001
*Tetramelaschloroleucus*	Westberg 10–001 (S)	KX512938	NA	KX512875	KX529006
*Te.geophilus*	**20-67496 (KUN)**	OL467354	OL467291	OL444785	**OM925563**
*Te.pulverulentus*	Nordin 6368 (UPS)	KX512940	KX512983	KX512860	KX528990
*Thelommamammosum* 1	Tibell 23775 (UPS)	KX512942	KX512954	KX512888	KX529016
*Th.mammosum* 2	Hernández et al. 2002 (UPS)	KX512943	KX512953	KX512851	KX529017
*Th.santessonii* 1	Nordin 4011 (UPS)	KX512944	KX512951	KX512889	NA
*Th.santessonii* 2	Nash 38262 (UPS)	KX512945	KX512950	KX512890	NA

ML analyses were performed with RAxML v8.2.12 (Stamatakis 2006). Bootstrap support values (MLBS) were estimated from the 70% majority rule tree of all saved trees obtained from 2000 non-parametric bootstrapping pseudo-replicates. BI analyses were performed with MrBayes v3.2.7 (Ronquist et al. 2012) running for 2 million generations. The trees were sampled every 100 generations and the first 25% of the trees were discarded as burn-in, since the average SD of split frequencies had converged at the step of 20% of the total. Bayesian posterior probabilities (BPP) were obtained from the 95% majority rule consensus tree of all saved trees. The final trees were visualized in FigTree v1.4.0 (Rambaut 2012). The final matrices were submitted to TreeBASE: TB2: 29562 for nuITS and TB2: 29563 for the combined regions dataset.

## ﻿Results

**Figure 1.**
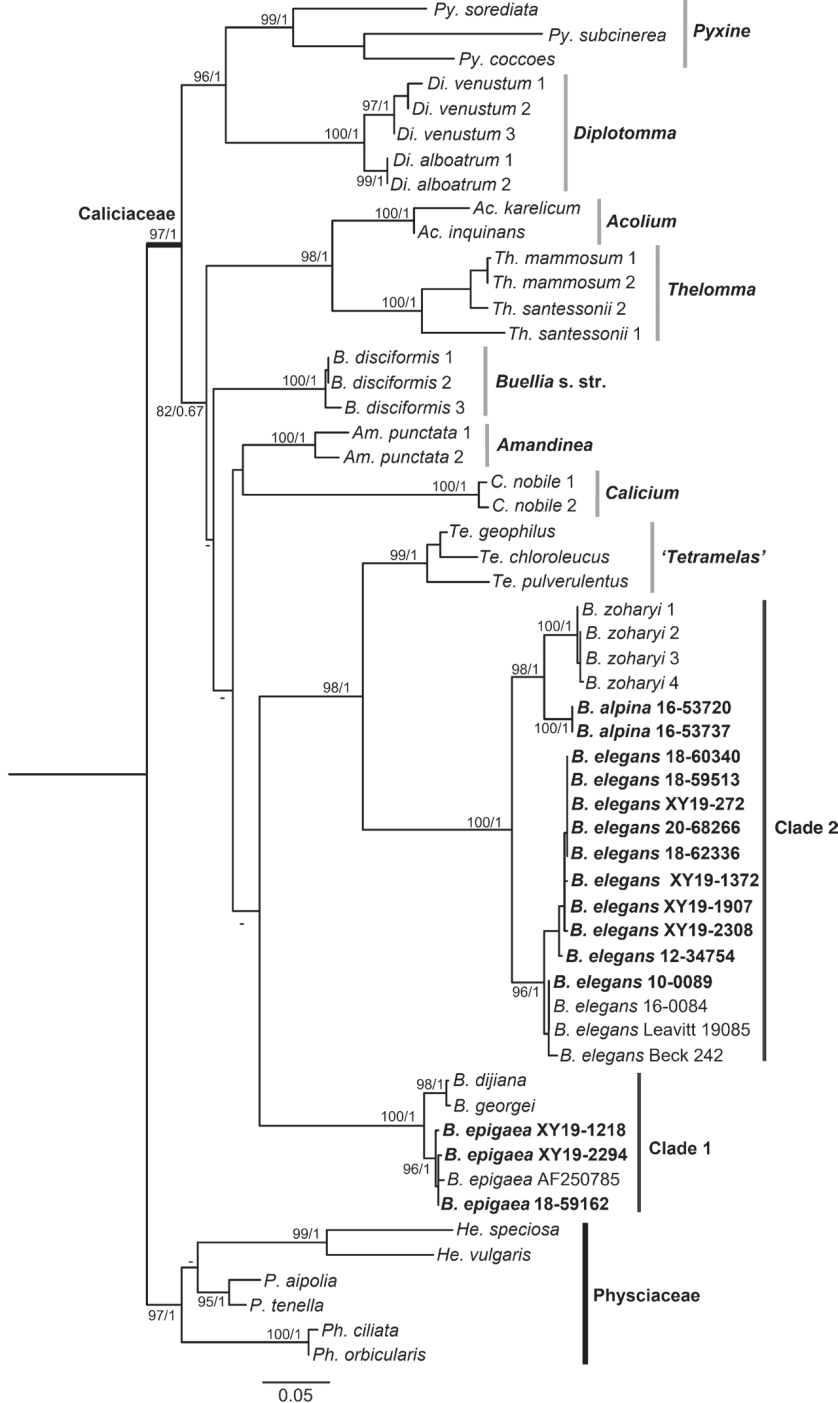
Phylogenetic relationships of Caliciaceae based on a Maximum Likelihood analysis of the nuITS matrix. Species positioned in clade 1 and clade 2 belong to the *Buelliaepigaea*-group. Maximum Likelihood bootstrap values and posterior probabilities are shown near the nodes. New species and records are shown in bold.

**Figure 2.**
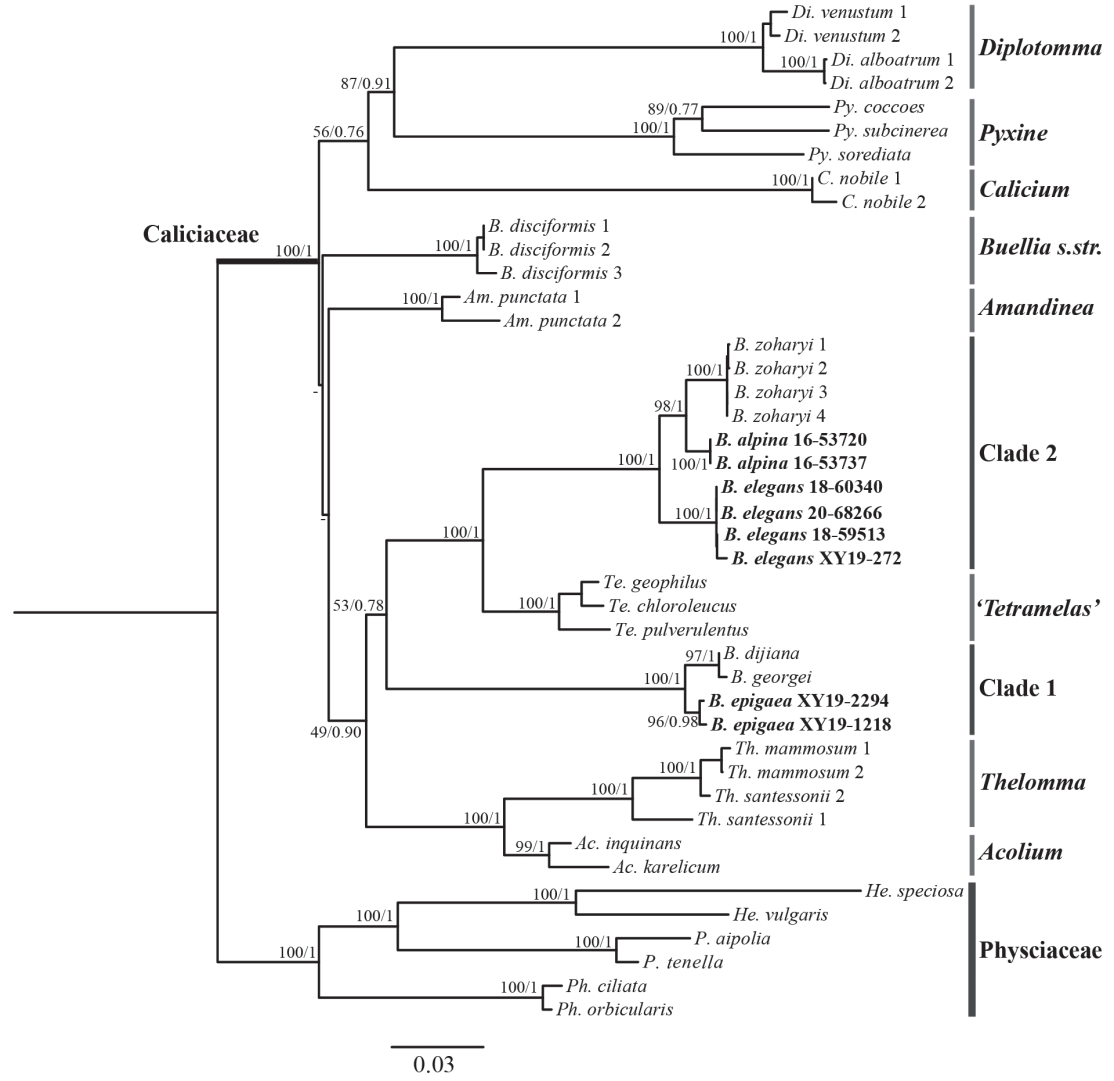
Phylogenetic relationships within Caliciaceae, based on a Maximum Likelihood analysis of a combined regions dataset (nuITS-nuLSU-mtSSU-β-tubulin). Species positioned in clade 1 and clade 2 belong to the *Buelliaepigaea*-group. Maximum Likelihood bootstrap values and posterior probabilities are shown near the nodes. New species and records are shown in bold.

The nuITS matrix (478bp) comprised 55 sequences including 15 newly generated sequences for new species and new records. The combined regions dataset (478bp for 43 nuITS sequences; 740bp for 27 mtSSU sequences; 899bp for 33 nuLSU sequences; 755bp for 23 β-tubulin sequences) comprised 126 terminals, including 31 newly generated sequences (Table 2). In the two phylogenetic analyses, eight representative monophyletic genera (*Acolium* (Ach.) Gray, *Amandinea*, *Buellia* s.str., *Calicium* Pers., *Diplotomma*, *Pyxine* Fr., *Tetramelas*, *Thelomma* A. Massal.) were selected from Caliciaceae Chevall. and six Physciaceae Zahlbr. species were selected as the outgroup. The results of the phylogenetic analyses showed that the species in the *Buelliaepigaea*-group formed two clades (Figs 1, 2).

In clade 1: *B.dijiana*, *B.georgei* and *B.epigaea* formed an independent clade with strong support (100% BS and 1.00 PP in Figs 1, 2). The specimens designated as *B.epigaea* (including one sequence from GenBank) clustered as a single lineage with high support (96% BS and 1.00 PP in Fig. 1). These specimens had identical morphological and chemical characters to those described for *B.epigaea*, and thus have been confirmed as a new record for China.

In clade 2: specimens here described as *B.alpina* formed a well-supported sister clade to *B.zoharyi* (100% BS and 1.00 PP in Figs 1, 2). These two species are distinctively different in their anatomical and chemical characteristics. We therefore recognize *B.alpina* as a new species within *Buelliaepigaea*-group. The collections designated as *B.elegans* also formed a highly supported monophyletic lineage, clustering with sequences downloaded from GenBank (96% BS and 1.00 PP in Fig. 1). This constitutes the first record of *B.elegans* from China. These three species (*B.zoharyi*, *B.alpina* and *B.elegans*) formed a monophyletic clade with strong support (100% BS and 1.00 PP in Figs 1, 2). Clade 2 was sister to the genus *Tetramelas* (previously included in *Buellia* s.l.), which also contains alpine terricolous species.

## ﻿Discussion

Although species in *Buelliaepigaea*-group share common characters, there are still additional diagnostic traits which could be used to distinguish between species within this group. The monophyletic clade 1 is formed by *B.dijiana* and *B.georgei*, together with *B.epigaea*. These three species share the characters of having no distinct marginal lobes and lacking atranorin. Within clade 1, only *B.georgei* has effigurate thalli; it also has short marginal lobes which often form rosettes. Both *B.georgei* and *B.dijiana* contain arthothelin acid and were described from Australia. However, their habitat differs: *B.georgei* occurs primarily on soft limestone or calcareous outcrops but never directly on calcareous soil, whereas *B.dijiana* is present on soil in open mallee vegetation (Trinkaus et al. 2001). In contrast, *B.epigaea* lacks secondary metabolites and could be reliably recognized by its crusty thallus, which is often uneven to wrinkled.

**Figure 3.**
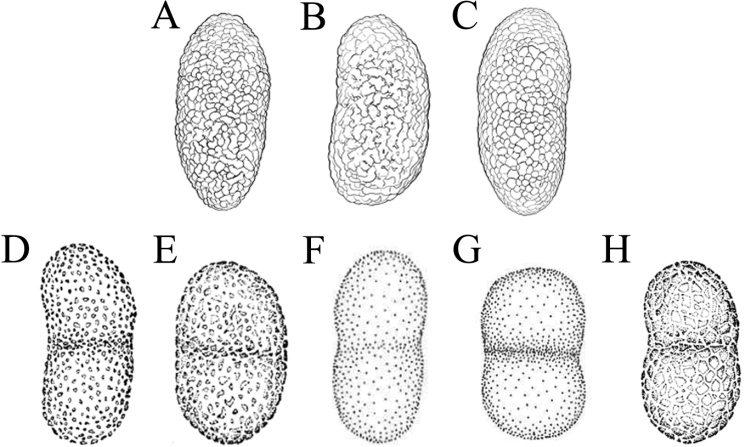
Ornamentation of ascospores **A***Buelliaalpina***B***Buelliaelegans***C***Buelliaepigaea***D***Buellialobata***E***Buelliadijiana***F***Buelliaasterella***G***Buelliazoharyi***H***Buelliageorgei* (**A–C** were drawn by Qiu Yi Zhong **D–H** are from Trinkaus and Mayrhofer 2000; Trinkaus et al. 2001).

**Figure 4.**
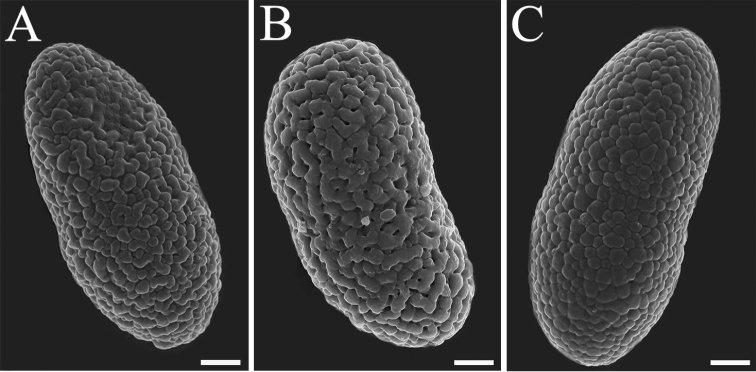
Ornamentation of ascospores (6000× magnification photograph under scanning electron microscope) **A***Buelliaalpina***B***Buelliaelegans***C***Buelliaepigaea* Scale bars: 2 µm (**A–C**).

We propose a new species: *Buelliaalpina*. It was clustered with *B.zoharyi* and *B.elegans* within clade 2. The common features of clade 2 are: having slim effigurate thalli covered with granulose pruina, obvious marginal lobes and always containing atranorin. The most distinctive features of the new species *B.alpina* are: heavily white pruinose apothecia and four-spored asci. *B.elegans* is similar to *B.zoharyi* in its external morphology. However, *B.elegans* can still be reliably distinguished from *B.zoharyi*, based on the ornamentation of ascospores. *B.elegans* has a loosely regulate surface (Fig. 3B and Fig. 4B), whereas the surface of *B.zoharyi* is microfoveate (Fig. 3G). In addition, the two species differ in their secondary metabolites: *B.zoharyi* contains atranorin, stictic acid and norstictic acid, while *B.elegans* has four chemotypes (Trinkaus and Mayrhofer 2000). One of these chemotypes (atranorin and 2’-O-methylperlatolic acid) is widely distributed in Asia, and was detected in most of the specimens from Yunnan, Qinghai and Xizang Provinces, China.

In addition to the species discussed above, *Buelliaepigaea*-group also contains the species *B.asterella* and *B.lobata*. These have not been included in this phylogenetic study due to the lack of available sequences. Morphologically, *B.asterella* and *B.lobata* are similar to *B.alpina* in their possession of four mature ascospores within each ascus (Trinkaus and Mayrhofer 2000; Trinkaus et al. 2001). *B.alpina* differs from these two species by its heavily white pruinose apothecia, granular pruina on the thallus surface and atranorin content. Furthermore, *B.alpina* has *Callispora*-type ascospores (with lateral (subapical) thickening, always with tapering ends). However, *B.asterella* and *B.lobata* both have fine pruina on their thallus surface and *Buellia*-type ascospores (lacking distinct wall thickening). *B.asterella* contains atranorin, norstictic acid and trace quantities of stictic acid, whereas *B.lobata* contains atranorin and thuringione.

The *Buellia* species in this study have all been classified as belonging to the *Buelliaepigaea*-group, based on their terricolous habitat and distinct morphological characters of white and effigurate thalli. However, the phylogenetic trees in this study suggest that this group is not monophyletic. Thus, the previous definition of *Buelliaepigaea*-group may be artificial, without support from molecular data. Phylogenetic study of both a single region (nuITS) and combined regions (nuITS-nuLSU-mtSSU-β-tubulin) showed that both clades do not group together. Therefore, the fundamental concept of the *Buelliaepigaea*-group requires further research, including additional samples from across its global distribution.

In conclusion, species of *Buelliaepigaea*-group share common characters which can be reliably recognized. There are distinct morphological and chemical differences which could be used to distinguish between different species in this group. Ornamentation of ascospores is a useful character by which to distinguish species in *Buelliaepigaea*-group (Figs 3, 4; Table 3).

**Table 3.** Key characteristics of the *Buelliaepigaea*-group.

Species	Thallus	Apothecia	Exciple	Spores	Ornamentation of spores	Major chemistry	Pycnidia	Parasitic fungi
*B.alpina*	effigurate, lobes linear, closely aggregate; covered with granulose pruina	flat, margin wavy and irregular	*dispersa*-type	*Callispora*-type; four-spored	densely rugulate, resulting in rough surface	atranorin	not seen	not seen
*B.asterella*	effigurate, lobes short and connected; surface with fine pruina	convex	*aethalea*-type	*Buellia*-type; often four well developed spores	microfoveate	atranorin, stictic acid, norstictic acid	rare	not seen
*B.dijiana*	not effigurate, crustose to granulose-squamulose; surface with fine pruina; dispersive	soon irregularly convex	*aethalea*-type	*Buellia*-type	warty, microrugulate	arthothelin	filiform conidia	rare
*B.elegans*	effigurate, lobes short to slender, multi-forked; covered with granulose pruina	flat to convex	*dispersa*-type	*Buellia*-type	Loosely rugulate, resulting in rough surface	a) atranorin; b) atranorin and 2'-O-methylperlatoric (in Asia)	not seen	common
*B.epigaea*	not effigurate, crusty, uneven to wrinkled; surface with fine pruina	flat	*aethalea*-type	*Callispora*-type	surface densely areolate and rough	no secondary metabolite	rare	rare
*B.georgei*	effigurate, marginal lobes short and often forming rosettes; surface with white granulose pruina	flat or sometimes slightly convex	*aethalea*-type	*Buellia*-type	surface densely areolate and rough	arthothelin	filiform conidia	common
*B.lobata*	effigurate, marginal lobes distinct but short, the tips of lobes dark; surface with lightly fine pruina	apothecia disc below margin	*aethalea*-type	*Buellia*-type; often four well developed spores	warty, microrugulate	arthothelin, thuringione	filiform conidia	common
*B.zoharyi*	effigurate, lobes obvious; covered with granulose pruina	flat to convex	*dispersa*-type	*Buellia*-type	microfoveate	atranorin, norstictic acid, stictic acid	common	rare

### ﻿Taxonomy

#### ﻿ Buellia alpina

Taxon classificationFungi

Xin Y. Wang & Li S. Wang sp. nov.

DC6E31F7-9A7B-522B-910D-5233C6476804

843376

Fig. 5

##### Diagnosis.

The species is distinguished from its closest relatives *B.elegans* and *B.zoharyi* by its linear lobate thallus, heavily pruinose apothecia and lobes, *Callispora*-type ascospores and four-spored asci.

##### Type.

China. Xizang Prov.: Lasa Ci., Namucuo Nature Reserve, on soil beside a lake, 30°46'46"N, 90°52'24"E, alt. 4730 m, 28 Sep. 2016, L.S. Wang et al. 16-53720 (KUN-Holotype; SDNU-Isotype).

##### Description.

Thallus effigurate, lobate and linear, lobes tightly aggregated, 0.5–1.5 mm wide, prothallus absent; upper surface white to grayish white, dull, covered with granulose pruina; medulla white, non-amyloid (I–). Apothecia sparse to dense, sometimes aggregate, adnate to the thallus, lecideine, margin covered with white pruina which resemble lecanorine apothecia; disc black, roundish, (0.3–)0.5–1.4(–1.6) mm in diam., heavily pruinose, roundish when immature, marginal part becoming wavy and irregular when mature; margin persistent; exciple *dispersa*-type (Bungartz et al. 2007), dark brown, without aeruginose pigments (HNO_3_–); epihymenium brown to dark brown; hymenium hyaline, 80–100 µm tall, without oil droplets, paraphyses simple to moderately branched, apically swollen, with a brown pigment cap; hypothecium dark brown; asci oval-clavate, *Bacidia*-type, four-spored; spores 1-septate, hyaline when young, turning brown when mature, *Callispora*-type (Bungartz et al. 2007), ellipsoid, with tapering ends, proper septum narrow, not thickening during spore ontogeny, (13–)15–20(–22) × (6–)7–9(–10) µm. Pycnidia not seen.

##### Chemistry.

Thallus K+ yellow, C–, PD–, UV–, medulla I–; containing atranorin.

##### Distribution and ecology.

This species is mainly distributed in alpine meadows of the Tibetan Plateau, growing on soil within meadows, between elevations of 4700–5000 m.

##### Etymology.

The epithet “*alpina*” refers to the alpine distribution of this species.

##### Note.

This new species could be distinguished from all other *Buellia* species by its linear lobate thallus, covered with granulose pruina, black lecideine apothecia with heavy whitish pruina, four-spored asci and its alpine distribution. It might be misidentified as subsquamulose or subfoliose species of *Squamarina* Poelt, but could be distinguished by the white thickened edges and hyaline simple ascospores.

**Figure 5.**
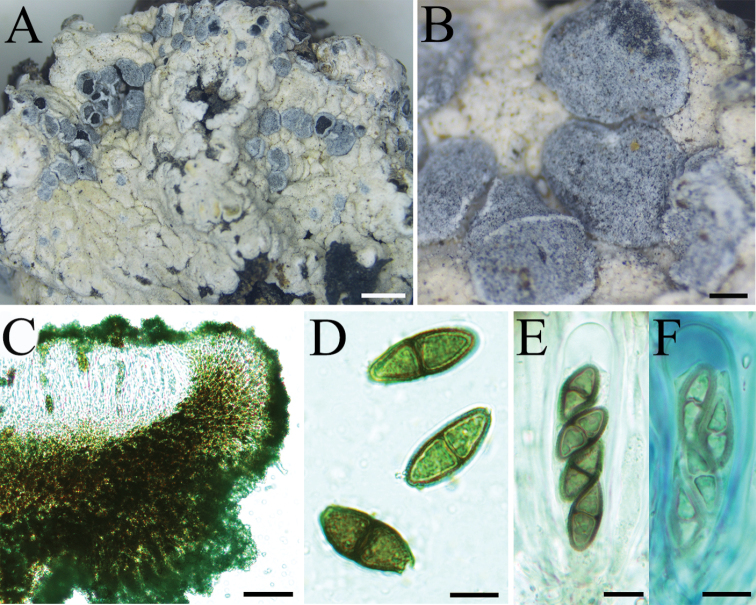
Morphology of *Buelliaalpina* (16-53720 KUN) **A** thallus on soil within meadow **B** black lecideine apothecia covered with white pruina **C** the section of apothecium, exciple *dispersa*-type **D** ascospores with 1-septate, *Callispora*-type, with tapered ends **E** mature ascus containing four spores, *Bacidia*-type **F** young ascus containing four spores. Scale bars: 2 mm (**A**); 0.5 mm (**B**); 50 µm (**C**); 10 µm (**D–F**).

##### Specimens examined.

China. **Xizang Prov.**: Lasa Ci., Namucuo Nature Reserve, on soil beside a lake, 30°46'46"N, 90°52'24"E, alt. 4730 m, 28 Sep. 2016, L.S. Wang et al.16-53737.

#### ﻿ Buellia elegans

Taxon classificationFungi

Poelt, Nova Hedwigia 25(1–2): 184–186 (1974)

4D597B7C-0C05-5F4C-B0D3-D2660F6D9AFE

Fig. 6

##### Type.

Italy. Ad terram calcaream supra Clavennam (Madèsimo), Anzi M. (M! -Holotype).

##### Description.

Thallus effigurate with distinct marginal lobes slim, 0.5–1 mm wide, the edge usually separated from the substrate and clearly foliaceous, thallus radiate, 1–2 cm in diam., prothallus absent; upper surface white, dull, usually covered with granular pruina; the upper cortex about 20 µm thick, with granular crystals, and the lower surface light brown to white, without cortex; medulla white, without calcium oxalate crystals. Apothecia sparse, lecideine; disc and margin black, sometimes lightly pruinose, roundish, 0.3–1.0 mm in diam., immersed and smooth when young but adnate and convex when mature; margin persistent; exciple thick, *dispersa*-type, without aeruginose pigments (HNO_3_–); epihymenium brown to dark brown; hymenium hyaline, 70–90 µm tall, without oil droplets, paraphyses simple to moderately branched, apically swollen, with a brown pigment cap; hypothecium dark brown; asci oval-clavate, *Bacidia*-type, eight-spored; spores 1-septate, hyaline when young, turning brown when mature, *Buellia*-type, ellipsoid, not thickening during spore ontogeny, 15–22 × 7–10 µm. Pycnidia not seen.

**Figure 6.**
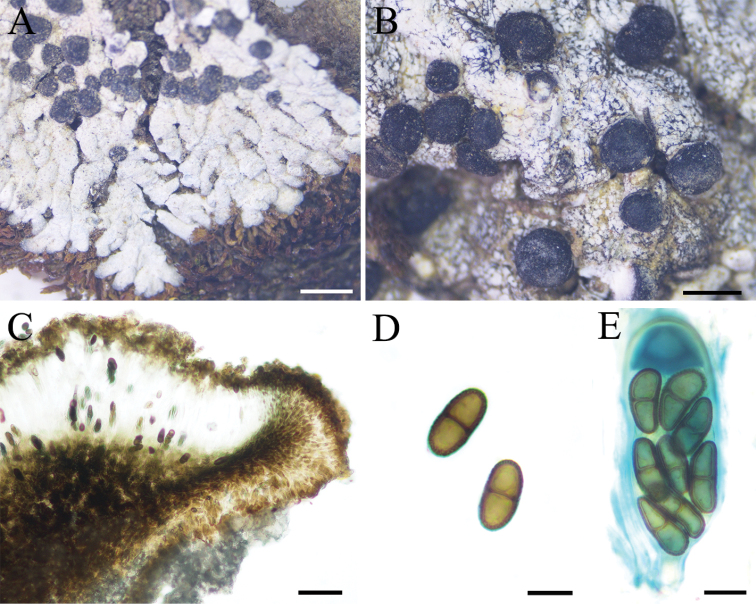
Morphology of *Buelliaelegans* (16-51770 KUN) **A** thallus on soil within meadow **B** lecideine apothecia **C** the section of apothecium, exciple *dispersa*-type **D** ascospores with 1-septate, *Buellia*-type **E** mature ascus containing eight spores, *Bacidia*-type. Scale bars: 2 mm (**A**); 1 mm (**B**); 50 µm (**C**); 10 µm (**D, E**).

##### Chemistry.

Thallus K+ yellow, C–, KC–, PD–, UV+ yellow, medulla I–; containing atranorin and norstictic acid (trace) or atranorin and 2’-O-methylperlatolic acid.

##### Distribution and ecology.

This species is mainly distributed in open and dry soil or soil over rock or within meadows between elevations of 1400–4730 m. This species has been recorded in Asia, Afghanistan, Europe and North America (Thomson, 1997). In China, it is mainly distributed in Gansu, Ningxia, Qinghai, Xizang and Yunnan Provinces.

##### Note.

This is a new record for China, and is unique among species of *Buellia* due to its effigurate thallus, marginal lobes linear and slim, branched near the tips. It resembles folicolous species of *Physconia* Poelt, but could be differentiated by its slim lobes and lack of lower surface. It has a wide distribution across the Tibetan Plateau, especially in arid deserts and meadows. Four chemotypes of the species were previously reported (Trinkaus and Mayrhofer 2000). Only two chemotypes have been detected in Chinese materials: atranorin and 2’-O-methylperlatolic acid account for the majority, atranorin and norstictic acid (trace) constitute only a small proportion.

##### Selected specimens examined.

China. **Gansu Prov.**: Jiayuguan Ci., Xigou, mineral, on soil, 39°39'34"N, 97°56'15"E, alt. 2198 m, 28 May 2018, L.S. Wang et al. 18-59611; Yumen Ci., meadow along the route from Yumen to Yuerhong, on soil, 39°57'45"N, 96°39'23"E, alt. 2395 m, 27 May 2018, L.S. Wang et al. 18-59513. **Ningxia Prov.**: Zhongwei Ci., Shanpotou, Mengjiawan, on soil, 37°36'12"N, 104°55'06"E, alt. 1403 m, 18 Sep. 2010, D.L. Niu et al. 10-0089. **Qinghai Prov.**: Wulan Co., desert along the route from Wulan to Delingha, on soil, 37°02'08"N, 98°12'29"E, alt. 3072 m, 20 May 2018, L.S. Wang et al. 18-58303; Dulan Co., Xiangjia Vil., on sandy rock, 36°00'53"N, 97°44'36"E, alt. 3056 m, 15 Sep. 2020, L.S. Wang et al. 20-68266. **Xizang Prov.**: Dazi Dis., Bangdui Vil., on soil, 29°44'06"N, 91°24'55"E, alt. 3709 m, 16 Jul. 2019, L.S. Wang et al. 19-64615; Basu Co., beside Ranwu Lake, on soil over rock, 29°23'34"N, 96°50'20"E, alt. 3901 m, 15 Jul. 2019, X.Y. Wang et al. (XY19-278; XY19-272); Geji Co., beside S301 road, on soil, 32°14'47"N, 82°10'27"E, alt. 4514 m, 21 Jul. 2019, L.S. Wang et al. 19-63808; Bomi Co., along the route to Basu Co., on soil, 29°40'31"N,96°12'38"E, alt. 2920 m, 10 Nov. 2018, L.S. Wang et al. 18-62336; Langkazi Co., Simila Mt., on soil over rock, 28°50'37"N, 89°51'54"E, alt. 4343 m, 24 Jul. 2019, X.Y. Wang et al. XY19-1372; Sangri Co., Sangri Town, on soil over rock, 29°17'29"N, 92°05'30"E, alt. 3595 m, 30 Jul. 2019, X.Y. Wang et al. (XY19-1899; XY19-1907); Jangzi Co., Simila Mt., on soil over rock, 28°50'30"N, 89°51'48"E, alt. 4223 m, 24 Jul. 2019, X.Y. Wang et al. XY19-2308; Jiangda Co., Kakong Vil., on soil over rock, 31°20'22"N, 98°08'01"E, alt. 3785 m, 23 Sep. 2020, L.S. Wang et al. 20-68931. **Yunnan Prov.**: Deqin Co., Benzilan Vil., on soil over rock, 28°10'27"N, 99°22'53"E, alt. 2007 m, 26 Sep. 2020, L.S. Wang et al. 20-69241; Deqin Co., Benzilan Vil., beside JinSha river, on soil, 28°11'36"N, 99°21'08"E, alt. 2108 m, 19 Aug. 2018, L.S. Wang et al. 18-60340; Deqin Co., Benzilan Vil., on soil, 28°13'38"N, 99°19'20"E, alt. 2110 m, 3 Jul. 2012, L.S. Wang et al. 12-34754.

#### ﻿ Buellia epigaea

Taxon classificationFungi

(Pers.) Tuck. Gen. lich.: 185 (1872)

E87C9BB5-A2E4-5C9F-B400-C46DD85552A1

Fig. 7

##### Type.

Germany. Hesse, ad terram inter muscos non procul a Monte Meissner, 1794, Persoon (H-Ach-Isotype, not seen).

##### Description.

Thallus terricolous, tightly attached to the substrate, upper surface white or greyish white, usually with white fine pruina, thallus crusty, uneven to wrinkled, 0.2–1 mm thick, prothallus absent; the upper cortex 60–150 µm thick, with granular crystals, pith completely interspersed with Ca oxalate crystals; medulla white. Apothecia sparse to dense, lecideine, but usually surrounded by a thalline collar (pseudolecanorine); disc black, always with finely white pruina, roundish, 0.5–1.0 mm in diam., mostly flat, rarely slightly convex; young apothecia immersed and margin with finely white pruina breaking out broadly, mature apothecia adnate and margin absent or not obvious; exciple *aethalea*-type, up to 50 µm thick, without aeruginose pigments (HNO_3_–); epihymenium brown to dark brown; hymenium hyaline, 60–80 µm tall, without oil droplets, paraphyses simple to moderately branched, apically swollen, with a brown pigment cap; hypothecium hyaline to light brown, up to 100 µm high; asci oval-clavate, *Bacidia*-type, eight-spored; spores 1-septate, hyaline when young, turning brown when mature, with tapering ends, *Callispora*-type, often curved, not thickening during spore ontogeny, 12–20 × 6–10 µm. Pycnidia not seen.

**Figure 7.**
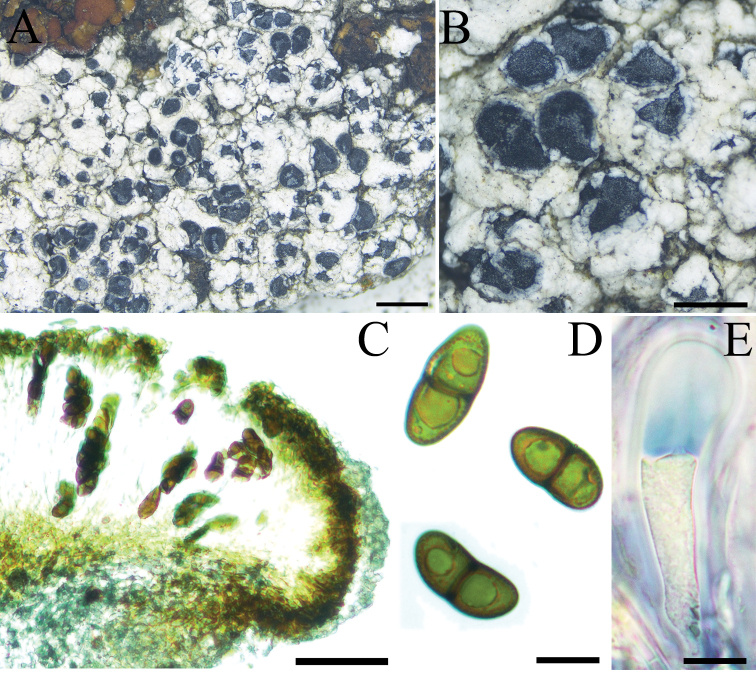
Morphology of *Buelliaepigaea* (XY19-2294 KUN) **A** thallus growing on the soil **B** lecideine apothecia with white pruina, surrounded by a thalline collar (pseudolecanorine) **C** the section of apothecium, exciple *aethalea*-type **D** ascospores with 1-septate, *Callispora*-type **E** ascus *Bacidia*-type. Scale bars: 2 mm (**A**); 1 mm (**B**); 50 µm (**C**); 10 µm (**D, E**).

##### Chemistry.

Thallus K–, C–, KC–, PD–, UV–; without secondary metabolites.

##### Distribution and ecology.

This species mainly occurs on open and dry soil, soil within meadows or on soil over rock, between elevations of 2300–4700 m. This species has been recorded in Asia, Europe and North America (Trinkaus and Mayrhofer 2000). In China, it is mainly distributed in Gansu, Qinghai and Xizang Provinces.

##### Note.

The species is a new record for China; it could be distinguished from all the other terricolous *Buellia* species reported in China by the combination of the following characteristics: thallus white crustose, uneven to wrinkled, always covered by finely white pruina, apothecia pseudolecanorine, the ornamentation of the ascospore surface densely areolate and rough, pycnidia rare, *Callispora*-type ascospores and absence of secondary metabolites. This species is close to *Tetramelas* species in phylogeny and similar in morphology, but could be distinguished by absence of the secondary metabolites 6-O-methylarthothelin or related xanthones, and pseudolecanorine apothecia covered with white pruina.

##### Selected specimens examined.

China. **Gansu Prov.**: Sunan Co., along the route from Linze to Sunan, on soil over rock, 38°52'26"N, 99°44'21"E, alt. 2294 m, 29 May 2018, L.S. Wang et al. 18-58766; Sunan Co., along the route from Linze to Sunan, on soil over rock, 38°52'47"N, 99°43'57"E, alt. 2296 m, 29 May 2018, L.S. Wang et al. 18-59699. **Qinghai Prov.**: Gonghe Co., meadow beside Qinghai Lake, on soil within meadow, 36°33'26"N, 100°28'45"E, alt. 3431 m, 18 May 2018, L.S. Wang et al. 18-59162. **Xizang Prov.**: Qushui Co., Niedang Vil., on soil, 29°30'24"N, 90°56'17"E, alt. 3527 m, 22 Jul. 2019, X.Y. Wang et al. XY19-1234; Qushui Co., Niedang Vil., on soil over rock, 29°30'22"N, 90°56'15"E, alt. 3624 m, 22 Jul. 2019, X.Y. Wang et al. XY19-1218; Langkazi Co., entrance to Karuola Glacier, on soil over rock, 28°53'54"N, 90°13'32"E, alt. 4774 m, 24 Jul. 2019, X.Y. Wang et al. XY19-2294.

### ﻿Key to species of *Buelliaepigaea*-group

**Table:** 

1	Thallus effigurate and marginal lobes long and obvious; containing atranorin	**2**
–	Thallus either not effigurate or effigurate but with marginal lobes short and closely aggregate; lacking atranorin	**4**
2	Ascus four-spored	***Buelliaalpina***
–	Ascus eight-spored	**3**
3	Spores large, up to 23 µm long, ornamentation of spores rugulate	***Buelliaelegans***
–	Spores smaller, less than 17 µm long, ornamentation of spores microfoveate	***Buelliazoharyi***
4	Thallus not effigurate	**5**
–	Thallus effigurate, usually with short lobes	**6**
5	Thallus crustose to granulose-squamulose; containing arthothelin	***Buelliadijiana***
–	Thallus crusty, uneven to wrinkled; lacking secondary metabolites	***Buelliaepigaea***
6	On rock; ascus eight-spored; marginal lobes forming rosettes	***Buelliageorgei***
–	On soil; usually four mature spores in each ascus	**7**
7	Containing atranorin and thuringione; ornamentation of spores warty, microrugulate	***Buellialobata***
–	Containing atranorin, norstictic acid and stictic acid (trace); ornamentation of spores microfoveate	***Buelliaasterella***

## Supplementary Material

XML Treatment for Buellia alpina

XML Treatment for Buellia elegans

XML Treatment for Buellia epigaea

## Supplementary materials

Supplementary material 1 Ornamentation of ascospores Data type Images Explanation note **Figure S1**. Ornamentation of ascospores (6000× magnification photograph under scanning electron microscope). A–D *Buelliaalpina*. Scale bars: 2 µm. **Figure S2**. Ornamentation of ascospores (6000× magnification photograph under scanning electron microscope). A–D *Buelliaelegans*. Scale bars: 2 µm. **Figure S3**. Ornamentation of ascospores (6000× magnification photograph under scanning electron microscope). A–D *Buelliaepigaea*. Scale bars: 2 µm. https://binary.pensoft.net/file/724248This dataset is made available under the Open Database License (http://opendatacommons.org/licenses/odbl/1.0/). The Open Database License (ODbL) is a license agreement intended to allow users to freely share, modify, and use this Dataset while maintaining this same freedom for others, provided that the original source and author(s) are credited.Min Ai, Li Juan Li, Fiona Ruth Worthy, An Cheng Yin, Qiu Yi Zhong, Shi Qiong Wang, Li Song Wang, Xin Yu Wang
