# Supplementary material for: ﻿Taxonomy of Buelliaepigaea-group (Caliciales, Caliciaceae), revealing a new species and two new records from China
Source: MycoKeys. 2022 Aug 5;92:45–62. doi: 10.3897/mycokeys.92.83939 (PMC9849091; doi:10.3897/mycokeys.92.83939)
Supplement: Supplementary material 1 — Ornamentation of ascospores [file mycokeys-92-045-s001.docx]

Appendix


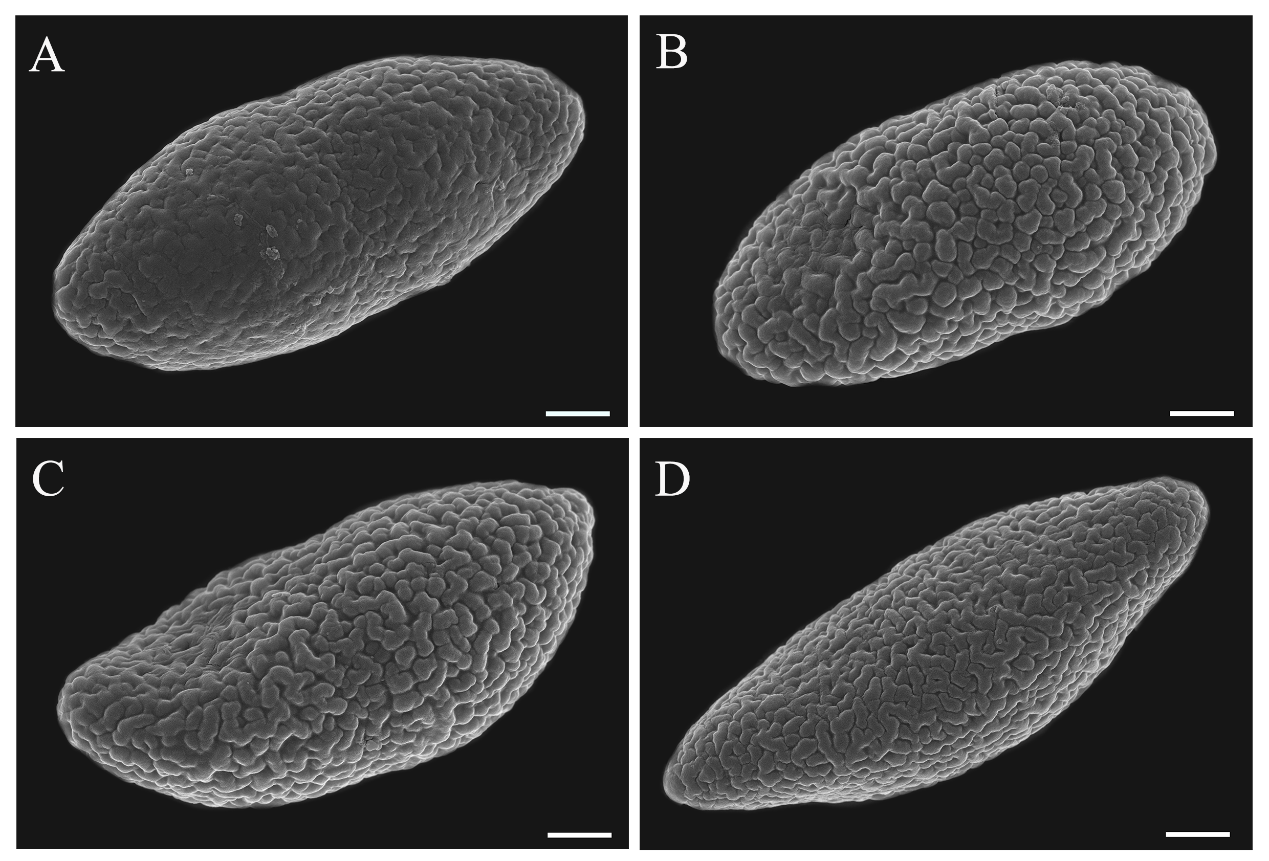


**Figure 1.** Ornamentation of ascospores (6000× magnification photograph under scanning electron microscope). A–D *Buellia alpina* Scale bars: 2 µm.
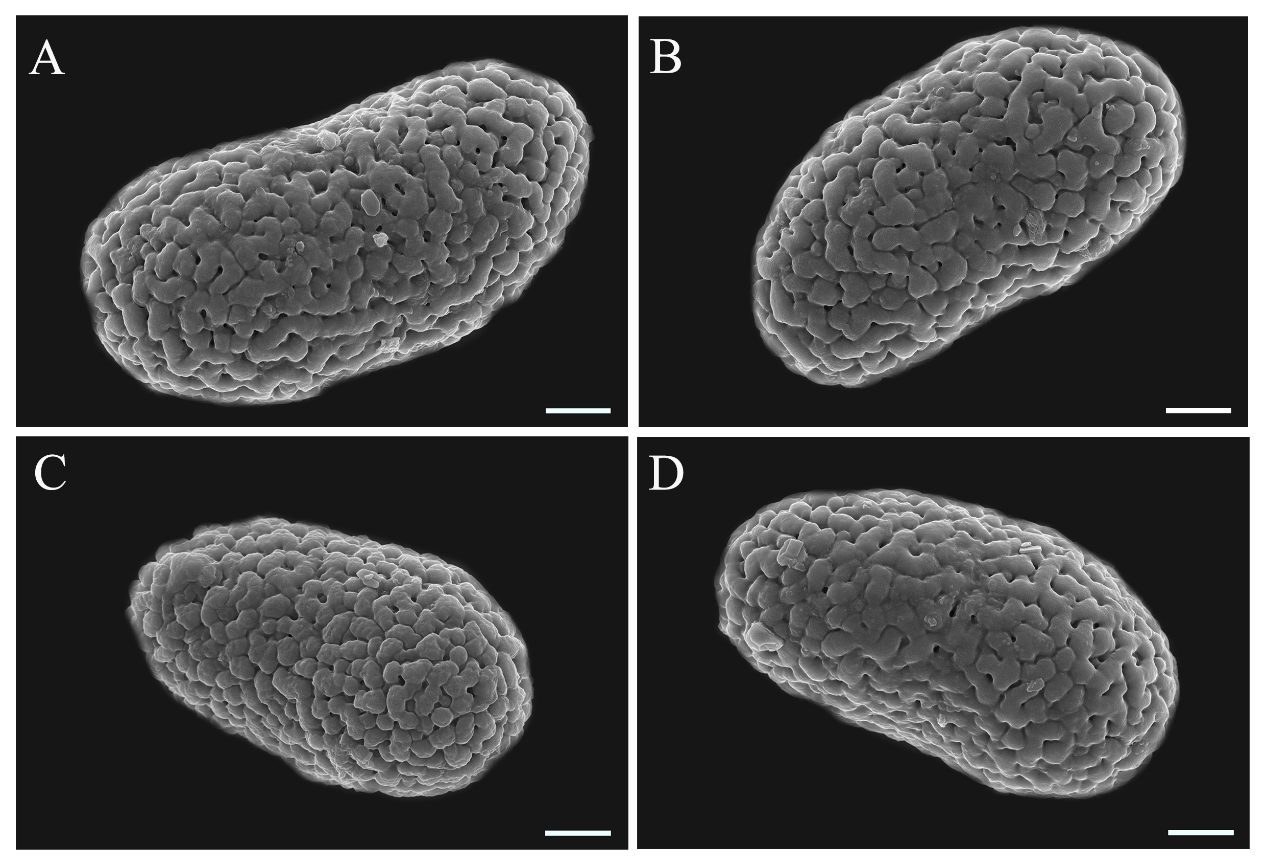


**Figure 2.** Ornamentation of ascospores (6000× magnification photograph under scanning electron microscope). A–D *Buellia elegans* Scale bars: 2 µm.


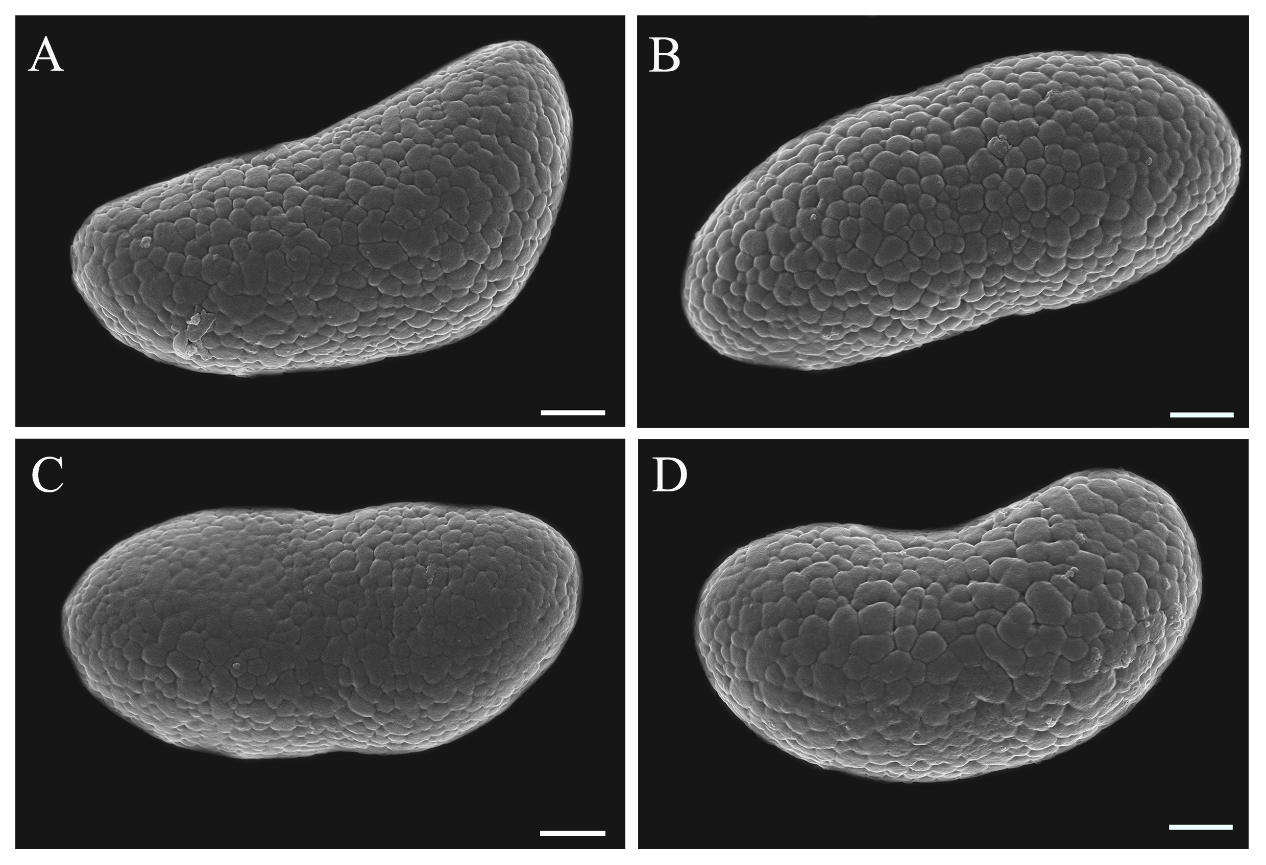


**Figure 3.** Ornamentation of ascospores (6000× magnification photograph under scanning electron microscope). A–D *Buellia epigaea* Scale bars: 2 µm.
